# Marathons and myasthenia gravis: a case report

**DOI:** 10.1186/s12883-018-1150-0

**Published:** 2018-09-18

**Authors:** Simone Birnbaum, Tarek Sharshar, Bruno Eymard, Marie Theaudin, Pierre Portero, Jean-Yves Hogrel

**Affiliations:** 1 Institute of Myology, GH Pitié-Salpêtrière (AP-HP), Bd de l’Hôpital, 75651 Paris Cedex 13, France; 2 Bioingénierie, Tissus et Neuroplasticité, EA 7377 Université Paris-Est Créteil Faculté de Médecine, 8 rue Jean Sarrail, 94010 Créteil, France; 3 Unité de Recherche Clinique Paris Île- de- France Ouest (URC PIFO), Raymond Poincaré Hospital, AP-HP, Garches, France; 4 Medical and Surgical Neurointensive Care Centre, Hospital Sainte Anne, Paris, France; 5 Laboratory of human histopathology and animal models, Institute Pasteur, Paris, France; 6 Université Paris Descartes, Paris, France; 7 Department of Neurology, CHUV, Rue du Bugnon, 46 1011 Lausanne, Switzerland

**Keywords:** Myasthenia gravis, Marathon, Auto-immune disease, Health, Endurance exercise

## Abstract

**Background:**

The cardinal symptoms of auto-immune myasthenia gravis are fatigue and weakness. Endurance events such as marathon running would seem incompatible with this chronic disease. Many patients stop sport altogether. There is limited literature of patients with auto-immune myasthenia gravis undergoing regular endurance exercise.

**Case presentation:**

We report the case of a 36-year-old female who began long-distance running whilst experiencing initial symptoms of myasthenia gravis. She was diagnosed with auto-immune myasthenia gravis and whilst advised to stop all sport, her way of fighting and living with this chronic and unpredictable disease was to continue running to maintain a healthy body and mind. Despite suffering from ocular, bulbar and localized limb fatigability, she managed to complete multiple marathons and achieve disease stability with cholinesterase inhibitors.

**Conclusions:**

Marathon and half-marathon running lead to distinct changes in mediators of inflammation in an exercise-dose-dependent manner. Despite symptoms of weakness and fatigue in certain muscles in myasthenia gravis, physical exertion remains possible and may not worsen symptoms as demonstrated in this case and recent studies. The immunomodulatory role of exercise could be considered in this case however this hypothesis remains to be confirmed in future studies with quantitative data.

## Background

Auto-immune myasthenia gravis (MG) is a chronic disease whereby dysfunction at the neuromuscular junction causes symptoms of fatigue and weakness [1]. Endurance events such as marathon running would seem incompatible with MG. Many patients stop sport altogether, finding activities of daily living to be challenging enough in themselves [2]. Even for the general public, marathon running is not practiced by a large majority, one must train regularly and have a high endurance capacity.

There are only three case studies combining sport and MG reported in the literature. Scheer et al. (2012) describe a 52 year old ultra-endurance athlete with mild MG (MGFA IIa), treated with 10 mg of prednisone who completed a 220 km ultra-marathon over 5 days [3]. Fatigue, leg weakness, dysphagia and breathing problems were reported, relieved by rest, shade and 60 mg of pyridostigmine every 90 min (max daily dose 720 mg). He began running 5 years prior to MG diagnosis and maintained a combined weekly running distance of 70 km. Stout et al. (2001) report a 26 year old athletic (baseball and weight lifting) student who suffered from extreme weakness and atrophy due to his MG, he was unable to do a single push-up at diagnosis [4]. Once stabilized he began exercising again at low levels which prevented further weakness but he struggled to regain force until undergoing a 15-wk upper and lower limb resistance training program, with oral creatine supplementation and he was able to make force gains (37% leg extension) and increase training volume (34–40% upper limb). Finally, Leddy et al. (2000) describe a 17 year old college football player with mild generalized MG (antibody-negative, 15% decrement RNS-EMG), treated with 60 mg prednisone every other day and 60 mg pyridostigmine q.i.d [5]. Following an initial period of weakness at the time of diagnosis, his strength returned to normal after 6 weeks and aside from a relapse associated with a period of non-compliance with medication, he became stable without treatment and was able to participate in full football practice.

Three recent uncontrolled trials have demonstrated benefits and tolerance of supervised physical activity (resistance or aerobic) programs for patients with stable MG [6–8]. Additionally, using the contralateral limb as a control, Lohi et al. found improvements in lower limb force following dynamic training and no deterioration nor negative side effects in a group of 11 subjects with mild MG [9]. A review article has suggested that risk factors for exacerbation of MG include prolonged exercise, running uphill and activity with stairs, however no specific data was provided to support these claims [10]. Currently there are no official guidelines regarding participation in sport and MG [11, 12].

## Case presentation

Here we report a 36-year-old female nurse, working full-time 12-h night shifts in a busy intensive care service, with generalized (MGFA IIb) auto-immune MG, symptomatic and dependent on cholinesterase inhibitors. Past medical history includes eczema in her teens, she is a carrier for sickle disease (sickle cell trait (SCT)) and she carried two pregnancies to term. Red blood cells have slightly reduced mean corpuscular volume and mean corpuscular hemoglobin concentration, 75.5 fl and 25.7 pg, respectively. She has no clinical symptoms of anemia and no specific treatment or monitoring. Surgical history includes 1 cesarean (2004), linea alba repair (2011) and breast implant (2014). She is a nonsmoker and does not drink alcohol. She is right-handed. She has a normal body mass index, 20.3 kg/m^2^, weighing 52 kg for 1.60 m.

Despite already experiencing abnormal weakness, she began running a year before being diagnosed with MG. Prior to running, she played amateur level basketball however this became incompatible with working night-shifts. MG diagnosis was based on clinical signs (right hand weakness - difficulty brushing teeth and hair, carrying light loads, cutting meat, a heavy head, nasal voice, ptosis, diplopia, dysphagia and difficulty masticating and articulating), serum auto-antibodies against nicotinic acetylcholine receptors (AChR) (> 100 nmol/l) and significant decrement on repetitive nerve stimulation (3 Hz) EMG (50% right trapezius, 24% right anconeus, 15% left anconeus, 43% tongue/mouth (CN V/XII)). Myasthenic muscle score (MMS) was 65/100. Initial treatment consisted of intravenous immunoglobulins (2 g/kg over 3 days (100 g)) and 60 mg pyridostigmine (t.i.d). Thoracic CT scan did not show thymoma but was in favour of thymic hyperplasia. Thymectomy was not performed as per the patient’s request.

**Fig. 1:**
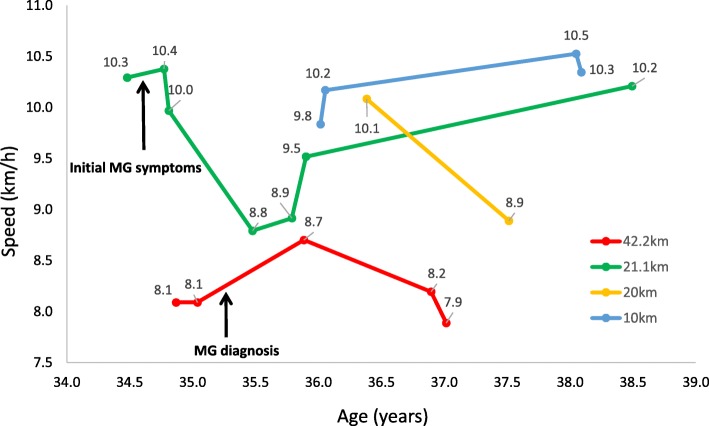
Running performance timeline

**Fig. 2:**
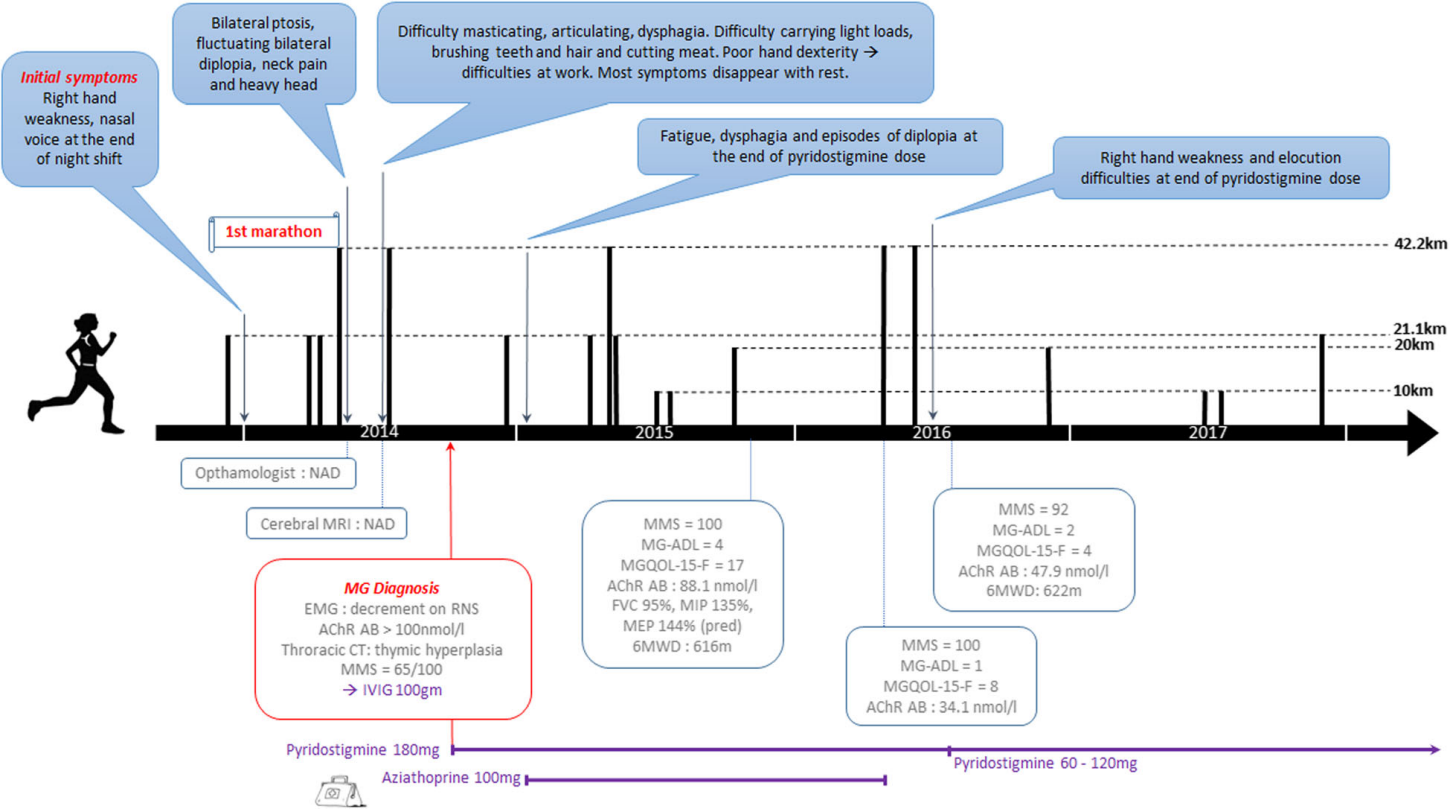
Diagnosis, clinical evaluation and running timeline

Despite the patient being informed at diagnosis that sport was contra-indicated, she kept running. Training consisted of 1–2 10 km weekly runs with the beginning being the most difficult. MG symptoms persisted including fatigue, dysphagia and episodes of diplopia at the end of pyridostigmine dose thus aziathoprine (100 mg) was introduced. She performed a half marathon 2 months later, followed by a full marathon and another half marathon. No major difficulty was experienced and performances significantly improved: 5h13mins (8.1 km/h) for a marathon pre-diagnosis to 4h51mins (8.7 km/h) post diagnosis (and treatment), Figs. 1 & 2.

On clinical evaluation 1 year after MG diagnosis, she had above average respiratory strength (MIP and MEP 135% and 144% of theoretical) and normal respiratory function (FVC 95% of theoretical). On maximal voluntary testing, limb strength was normal (knee extensors and elbow flexors: 128% and 101% of theoretical, respectively and right handgrip strength: 88%). Walking endurance was also considered normal (92% of theoretical 6MWD). The MMS suffered from a ceiling effect with the patient achieving the maximal score of 100 and a score of 4 on the MG-ADL due to episodes of dysphagia, dyspnea with effort and UL fatigue with brushing her teeth and hair. She reported recurrent MG symptoms including loss of hand dexterity (difficulty manipulating medication at work), bulbar symptoms such as dysphagia on her own saliva, a nasal voice and ocular symptoms (ptosis and diplopia). MG-specific quality of life (MGQOL-15-F) was reduced (17/60) particularly with regards to professional and social aspects, reflected in the WHO-QOL Bref with the social relationship and physical health domains being reduced, 11/20 and 13/20 respectively [13, 14].

She continued regular running without any particular changes in her MG, recurrent right hand weakness persisted and she experienced occasional ocular and bulbar symptoms primarily at the end of pyrodistigmine dose. Quality of life improved significantly over time (Fig. 2) and strength remained stable (knee extensors and elbow flexors: 128% and 100% of theoretical, respectively, right handgrip strength: 93%). She discontinued aziathoprine (treatment duration 14 months) and remained exclusively treated with cholinesterase inhibitors (60 mg, b.i.d).

## Discussion and conclusion

It is well known that MG does not affect all muscles to the same extent and whilst some muscles may clearly be affected, other muscles may function normally and can be trained without adverse effects. Thus, continuing activities and daily life seems important for maintaining “unaffected” muscles and for psychological benefits. With regards to physical activity a close link between endurance running and the immune system activity has been demonstrated [15]. During sustained physical activity, repeated muscle contractions stimulate the production of inflammatory cytokines such as Interleukin-6 (IL-6) by myocytes. IL-6 may act as an inhibitor to pro-inflammatory cytokines such as TNF-α which is a potent mediator of tissue damage [16, 17]. IL-6 also stimulates the production of IL-10 (an anti-inflammatory cytokine) and IL-1ra, generating an anti-inflammatory environment. Whether these effects can modify disease activity in MG currently remains unclear. These exercise-induced mediators of inflammation are susceptible to exercise dose, with greater increases seen following a marathon as compared to a half marathon, for example [16]. A disease-specific response has been suggested for the change in cytokine kinetics in relation to exercise [18].

Whilst evidence of exercise-induced change of cytokines remains sparse in MG, there is growing evidence of the presence of inflammation and modulation of the immune response by cytokines in MG pathogenesis [19, 20]. Certain cytokines are upregulated (IL-17, IL-21, IL-6, IL-10) and others downregulated (IL-4) compared to control subjects [19, 21–23]. A recent study evaluated serum IL-6 before and after a twice-weekly, 3 month training program in subjects with ocular or mild, generalized MG. Serum Il-6 levels remained unchanged [7]. However, circulating microRNAs (miR150-5p and miR21-5p) decreased [7]. These immuno-microRNAs have been suggested as new biomarkers in AChR MG, notably miR150-5p as a marker for disease severity [24, 25].

Another factor which warrants consideration here is the localized role of IL-6 in triggering satellite cells in the myogenic process [26]. Whilst research has demonstrated the role of IL-6 in muscle atrophy, this has predominantly involved pathological situations such as cancer cachexia and ageing [27] where there is chronic inflammation. When released at low concentration into satellite cell niches IL-6 could promote repair and regenerate skeletal muscle tissue [27]. It seems that IL-6 is deregulated in the muscles of MG patients, possibly due to AChR antibodies deregulating the IL-6 pathway [28].

In the case of this patient, weakness induced by MG mechanisms tends to affect the same muscles which were initially affected, regardless of increased exertion. Whether this patient has benefitted from protective effects of exercise or not is difficult to determine, however, this case study demonstrates that an endurance sport such as running marathons and MG are not mutually exclusive. The immunosuppressor treatment likely played an essential role in attenuating the autoimmune attack enabling the patient to improve and optimize her physical capacities. Clinical scores improved and remained stable and running performance improved and remained relatively consistent (Figs. 1 and 2). The level of antibodies against AChR decreased over time (Fig. 2) however this also coincided with immunosuppressor treatment.

In stabilized MG there are no clear arguments to fear adverse effects of physical activity and as well as positive psychological effects, patients will benefit from cardiovascular and muscular adaptions as well as possible immunomodulatory effects. It is important to consider each individual case and weigh up the benefits and risks and possibly put into place a supervised or specific return to sport program where necessary. As can be expected in the absence of disease, inter-individual variation is likely with strenuous physical exercise.

It seems important to discuss the possible consequences of exercise and sickle cell trait which are not currently clear in the literature. Case-control studies have found an increased prevalence of sudden death on exertion, often called “exercise collapse associated with sickle trait” however a direct causal relationship has not been confirmed [29]. It has been suggested that a combination of factors [30] such as high altitude or severe dehydration combined with intense physical activity could cause red blood cells to become deformed or sickled and cause complications such as rhabdomyolysis. This may be due to lower capillary density and tortuosity, reduced small vessels and a higher percentage of broader micro vessels [31]. Microvascular remodeling may be a compensatory mechanism to facilitate normal blood flow [29] which may be present in this patient considering the raised but not excessive elevation of CK following a marathon (data not presented). SCT may not be a completely benign carrier state nor a true disease entity but rather a potential risk factor which could be monitored. Maintaining physical fitness may be a protective agent by decreasing the endothelial activation through improvements in nitric oxide and antioxidant availability [32, 33]. Precautions would be the same as for exercise with MG i.e.; adequate hydration, control heat exposure and monitor exertion intensity [34].

This case report demonstrates that despite being in the first 2 years of MG disease onset in which exacerbations and instability are most common, and despite regular intense endurance running, the MG has remained stable. A growing body of literature suggests that regular physical exercise may lead to anti-inflammatory effects in chronic auto-immune diseases. The immunomodulatory role of exercise could be considered in this case study. However, further studies regarding the specific effects of exercise on disease activity in MG are necessary to confirm these speculations.

### Patient perspective

“I am myasthenic and marathonian, running allows me to take time for myself, to evacuate stress and to think. It’s a real therapy”.

## Abbreviations

6MWD: Six-minute walk distance
AChR: Acetycholine receptor
b.i.d: Twice a day
CN: Cranial nerve
EMG: Electromyogram
IL-6: Interleukin-6
MG: Auto-immune myasthenia gravis
MG-ADL: Myasthenia Gravis Activites of Daily Living score
MGFA: Myasthenia Gravis Foundation of America disease classification
MGQOL-15-F: Myasthenia Gravis 15 item French Quality Of Life scale
MIP/MEP: Maximal inspiratory pressure/Maximal expiratory pressure
MMS: Myasthenic Muscle Score
SCT: Sickle cell trait
t.i.d: Three times a day
TNF-α: Tumour Necrosis Factor alpha
UL: Upper limb
WHO-QOL Bref: World Health Organisation Quality Of Life short questionnaire
